# Hepatoid adenocarcinoma of the duodenal papilla with hepatic metastases: A case report and literature review

**DOI:** 10.3389/fonc.2022.948892

**Published:** 2022-08-08

**Authors:** Lu Han, Naiqing Ding, Li Li, Xiao Wei, Jing Hu, Baorui Liu, Xiaoping Qian

**Affiliations:** ^1^ Nanjing Drum Tower Hospital Clinical College of Traditional Chinese and Western Medicine, Nanjing University of Chinese Medicine, Nanjing, China; ^2^ The Comprehensive Cancer Centre of Drum Tower Hospital, Medical School of Nanjing University, Nanjing, China

**Keywords:** hepatoid adenocarcinoma of the duodenum, hepatoid adenocarcinoma, alpha- fetoprotein (AFP), immunohistochemical, hepatic metastases

## Abstract

Hepatoid adenocarcinoma of the duodenum is a rare special type of adenocarcinoma, featured by hepatocyte components in primary adenocarcinoma of the duodenum. It has the characteristics of high malignancy, invasiveness, rapid progress, and poor prognosis. An abnormal elevation of serum alpha-fetoprotein (AFP) may occur in most cases. The diagnosis is mainly based on pathological morphology. Here, we reported a case of hepatic adenocarcinoma of the duodenum. The middle-aged female patient had an ampulla mass at diagnosis and received radical pancreaticoduodenectomy. The postoperative pathology was stage IIIA duodenal adenocarcinoma. At 1 month after surgery, she had multiple intrahepatic metastases and retroperitoneal lymph node metastasis; the AFP level was 300 ng/ml at that time. As she refused target therapy, two cycles of capecitabine-oxaliplatin (XELOX) chemotherapy were performed. However, the AFP elevated from 300 to 1,931.90 ng/ml, and the disease progressed rapidly. Immunohistochemistry (IHC) of tissue samples from presurgical endoscopic ultrasound guided fine needle aspiration (EUS-FNA), surgery, and liver biopsy showed positive AFP staining. Combining the abnormal elevation of serum AFP and microscopic pathological morphology, this case is diagnosed as hepatoid adenocarcinoma of the duodenum with liver metastasis. The physical condition of this patient was too poor to receive follow-up treatment. She died of the rapid disease progression with an overall survival time of 161 days. Considering that in most patients with hepatoid adenocarcinoma the abnormal elevation of serum AFP occurs preoperatively and returns to normal postoperatively rather than normal before surgery and increased after surgery, the primary lesion is located in the stomach rather than the intestine, and the patients are more often older men rather than middle-aged women; this case is rare particularly. Therefore, reporting this case with complete case data may be helpful to further study, so as to improve the understanding of this special type of malignant tumor.

## Introduction

In 1970, Bourreille et al. (1) reported a case of hepatic metastasis from gastric adenocarcinoma with elevated serum alpha-fetoprotein (AFP) for the first time. In 1985, Ishikura et al. (2) proposed the term “hepatoid adenocarcinoma (HAC)”. HAC is a particular type of adenocarcinoma named for its similarity to the histological morphology and immunophenotype of hepatocellular carcinoma (HCC). It is a rare type with an estimated annual incidence rate of 0.58–0.83 per million people (2–4) and mostly happened in older men (5, 6). HAC is highly invasive and prone to liver metastasis, and prognosis is very poor, with an average overall survival rate of 10–18 months (7). HAC can be found in almost all malignant tumors of the digestive tract, especially in the stomach, accounting for about 63% of all HACs, possibly due to the fact that the stomach and liver are derived from the same part of the embryo (5, 8, 9). In addition, HAC can also occur in ovaries, lungs, bladder, etc. (10–12), and its onset is rare in the intestinal tract, especially in the duodenum. With the keywords hepatoid adenocarcinoma, duodenum, only three published papers were searched in the PubMed databases, and an article based on Surveillance, Epidemiology, and End Results (SEER) database found that the incidence of HAC of the gastrointestinal tract including the duodenum (excluding the stomach) in all HACs was only 5% (13). The scarcity of numbers reflects the rarity of HAC of the duodenum. Because the incidence of this special type of tumor is extremely low, its pathogenesis is not clear at present. Ishikura et al. (2) studied gastric HAC and found that the foregut derivatives of the primitive digestive tube were closely related to the production of AFP, which may be caused by the deviation in the cell differentiation process leading to tumor differentiation into hepatocytes. Sporadic cases of HAC originating from the intestinal tract have also been reported, most of which were accompanied by elevated serum AFP (14–16). Liming et al. (17) believe that one possibility is that the intestinal tract can produce a small amount of AFP during embryonic development, which promotes the appearance of tubular adenocarcinoma and hepatoid differentiation regions. In addition, it may be due to the existence of stem cells with bidirectional differentiation potential in the intestinal tract, which promotes glandular epithelium and hepatocyte differentiation (18, 19). Ziwei et al. (20) also believe that HAC cells resemble totipotent cells, which can proliferate in the early stage; this conjecture explains the generally poor prognosis.

The clinical manifestations of HAC patients are usually non-specific and mostly depend on the specific location of the primary tumor. For example, patients with colorectal cancer (CRC) may have abdominal pain, fever, or hematochezia. When a tumor occurs in the ampulla and nipple area of the duodenum, clinical manifestations such as yellow skin, yellow eyes, and yellow urine (such as in this case) can be seen. In most cases, patients have abnormally elevated serum AFP expression, but normal serum AFP expression (for example, in the early stage of diagnosis of this case) can also be seen.

Here, we introduce a middle-aged female patient diagnosed with hepatic adenocarcinoma of the duodenum with hepatic metastases; serum AFP was normal at the time of diagnosis but increased significantly with the progression of the disease. As the patient refused genetic testing and targeted therapy, she was treated with two cycles of XELOX chemotherapy and subsequently died due to rapid progression.

## Case presentation

### Diagnosis

**Figure 1:**
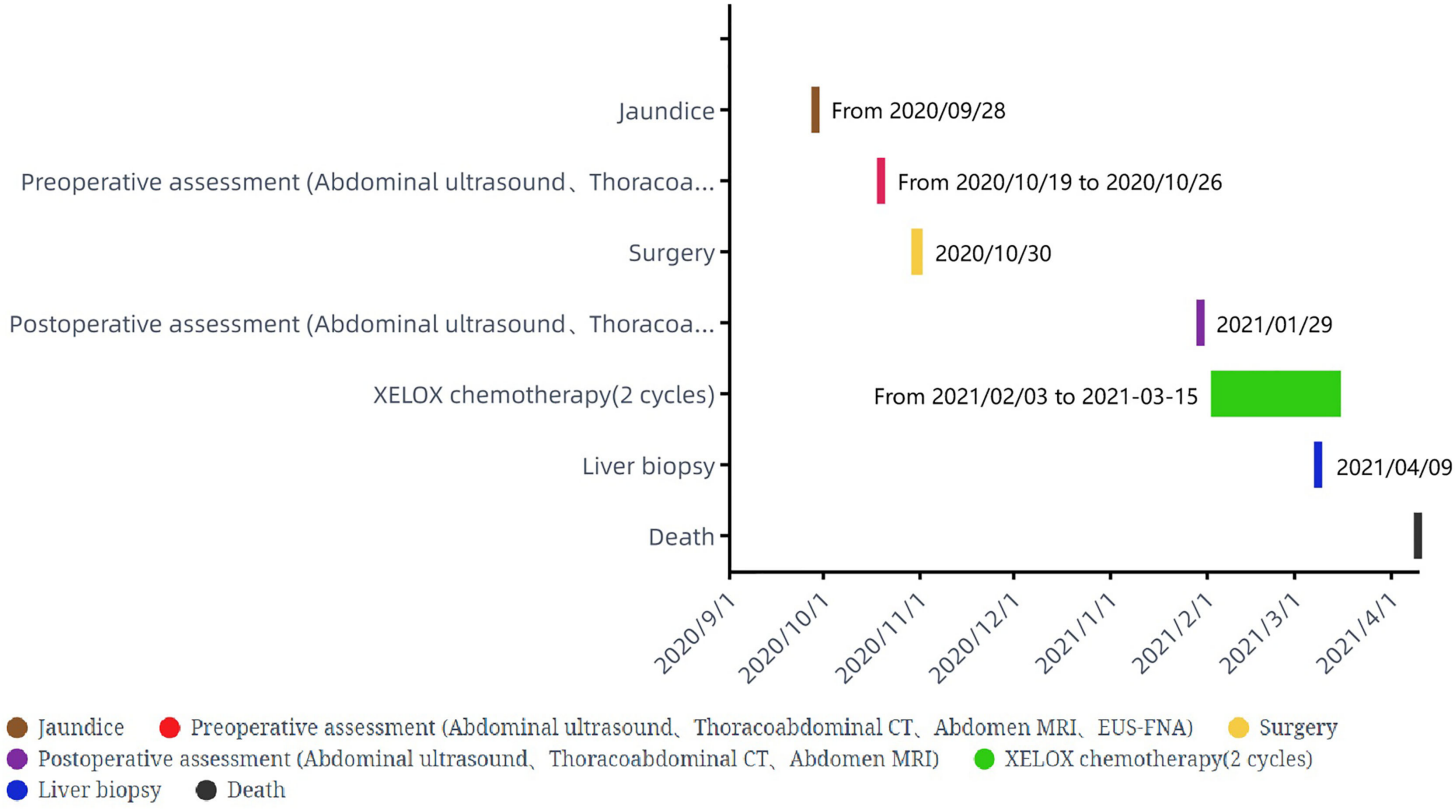
Time points corresponding to the diagnostic and therapeutic process.

Our patient is a 59-year-old woman with a history of hypertension and hyperglycemia for more than 15 years without previous history of alcohol consumption or smoking nor history of liver disease. She presented with intermittent fever without obvious inducement for 3 weeks (specific time points in **Figure 1**) with the highest temperature of 39°, accompanied by jaundice, occasional nausea, vomiting, and slight discomfort in the right upper abdomen. Biochemical examination revealed glutamic-pyruvate transaminase (ALT) levels of 198 U/L, glutamic-oxaloacetic transaminase (AST) 131 U/L, total bilirubin (TBil) 68 μmol/L, and direct bilirubin (DBil) 35.1 μmol/L. Blood routine examination revealed no obvious abnormalities. CT of the chest and abdomen showed no obvious active lesions in both lungs but gallbladder enlargement and dilatation of intrahepatic and extrahepatic bile ducts, common bile duct, and main pancreatic duct. MRI of the abdomen showed low biliary obstruction, and the obstruction level was located on the ampulla. Considering the possibility of inflammatory lesions, local hospitals gave anti-infection, fluid rehydration, liver protection, and other symptomatic treatment, but no obvious relief of symptoms.

**Figure 2:**
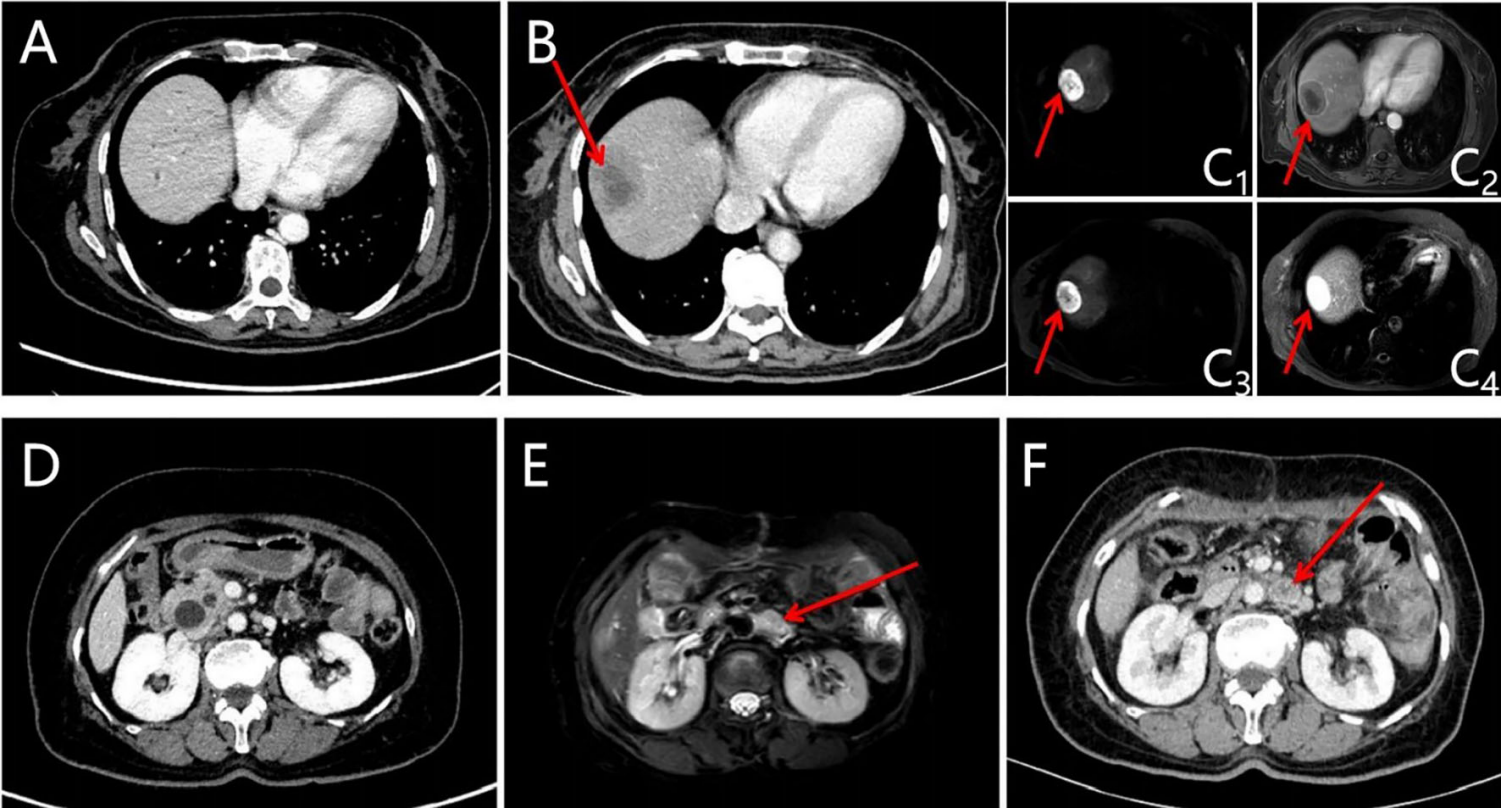
Images of the liver and retroperitoneal lymph node lesions were evaluated before and after the operation (new liver and retroperitoneal lymph node lesions after operation, shown by red arrows). **(A)** CT of liver before the operation; **(B)** CT of liver was evaluated at baseline after the operation; **(C)** MRI of the liver was evaluated at baseline after the operation (C_1_: DWI-b50; C_2_: T1WI; C_3_: DWI-b800; C_4_: T2WI); **(D)** CT of retroperitoneal lymph nodes before the operation; **(E)** CT of retroperitoneal lymph nodes was evaluated at baseline after the operation; **(F)** MRI of retroperitoneal lymph nodes was evaluated at baseline after the operation.

**Figure 3:**
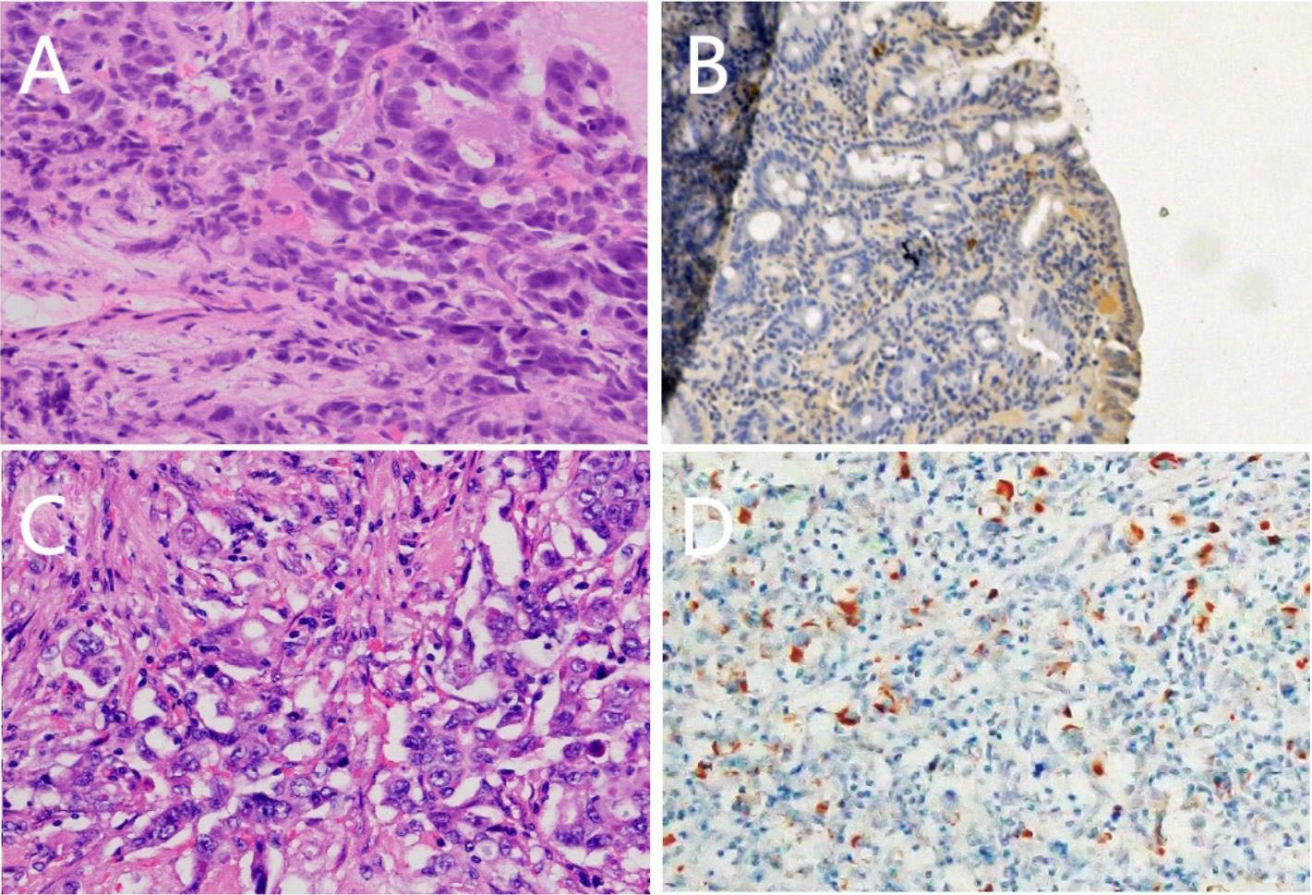
Pathological morphology **(A)** (HE ×400), expression of positive Alpha-fetoprotein(AFP) staining **(B)** (SP ×100) of tissue samples from presurgical endoscopic ultrasound-guided fine needle aspiration (EUS-FNA); Pathological morphology **(C)** (HE ×400), expression of positive Alpha-fetoprotein(AFP) staining **(D)** (SP ×100) of tissue samples fromsurgery.

Abdominal color ultrasound was performed at the general surgery department of our hospital on 19 October 2020. It showed that there is a mass in the ampullary region with dilation of intrahepatic and extrahepatic bile ducts and main pancreatic duct. Moreover, enlargement of the gallbladder was also shown. No obvious abnormality was observed in the liver. CT of pancreatic vascular reconstruction showed a mass in the ampulla, surrounded by enlarged lymph nodes, and was considered malignant. The intrahepatic and extrahepatic bile ducts and pancreatic ducts were dilated, the gallbladder was enlarged, and no obvious abnormalities of the liver and retroperitoneal lymph nodes were observed (**Figures 2A, D**). Tumor marker AFP was 5.00 ng/ml (normal range 0–10 ng/ml) (**Table 1**). The pathological result of EUS-FNA was moderately to poorly differentiated ampullary adenocarcinoma (**Figure 3A**); the immunohistochemistry (IHC) of EUS-FNA was AFP (+) (**Figure 3B**).

**Table 1:** Changes of Alpha-fetoprotein (AFP), Carcinoembryonic antigen (CEA) and Carbohydrate antigen 19-9 (CA19-9) levels at diagnosis, after the surgery, and after one cycle of chemotherapy.

Date	2020-10-18^a^	2021-01-29^b^	2021-03-04^c^
AFP (ng/ml)	5	330.9	1931.9
CEA (ng/ml)	54.31	22.56	25.62
CA19-9 (U/ml)	86.1	14.07	95.69

a Preoperative.

b after surgery.

c after one cycle of chemotherapy.

### Surgical intervention

After the patient was admitted to the hospital, the patient was evaluated with an Eastern Cooperative Oncology Group (ECOG) score result of 1. Physical examination revealed yellow skin and yellow sclera. The patient and family had no previous medical history of chronic viral hepatitis, bowel polyps, and inflammatory bowel disease (IBD). The patient underwent pancreaticoduodenectomy on 30 October 2020. Intraoperative exploration showed that there were no free ascites or metastatic nodules in the abdominal cavity, the liver had a normal size with cholestasis-like changes, and the gallbladder was swollen. A mass with a size of about 3 cm * 3 cm could be reached in the ampulla, and the texture was hard. Postoperative pathology (pancreaticoduodenectomy specimen) (**Figure 3C**) showed that the duodenal papilla had a poorly differentiated adenocarcinoma, the size of the mass was 4 cm * 2.5 cm * 1.2 cm, and the cancer tissue penetrated the duodenal muscle wall and invaded the pancreatic tissue. There is no definite cancer tissue invasion in the common bile duct. Vascular cancer embolus and perineural invasion can be seen. No cancer remains at the gastric margin, small intestinal margin, common bile duct margin, broken end margin of the pancreas, peripheral margin of the pancreas, and hook process margin of the pancreas. Cancer metastasis was found in 1/6 lymph nodes around the pancreas, and no cancer metastasis was found in 3 lymph nodes around the stomach, 1 lymph node around the intestine, and 2 lymph nodes around the common bile duct. Another 3 lymph nodes were sent, and no cancer metastasis was found. Pathological stage was IIIA (T4, N1, cM0); IHC: cancer cell expression CK(+), S100(-), Her2(Sto)(++), COX2(+), VEGFR2(-), EGFR(++), Ki67(about 60%+), P16(focal +), CD56(-), PD-1(-), PD-L1(SP142)(the tumor cells -, interstitial immune cells about 5%+), MLH1(+), MSH2(+), MSH6(+), PMS2(+), c-Met(+), CgA(-); AFP (+) (**Figure 3D**).

### Baseline assessment

One month after surgery, CT and MRI scan showed multiple hepatic masses (**Figures 2B, C**) and retroperitoneal enlarged lymph nodes (**Figures 2E, F**), which were considered metastases. The patient refused to accept gene testing and targeted therapy, so one cycle of XELOX 3-week regimen chemotherapy was performed according to National Comprehensive Cancer Network (NCCN) guidelines for CRC.

### Liver biopsy

**Figure 4:**
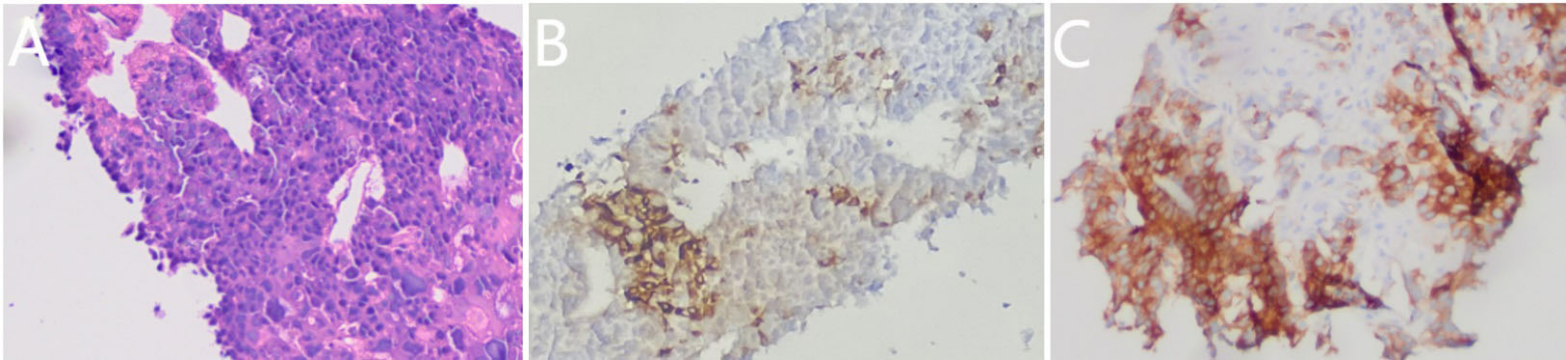
Pathological morphology **(A)** (HE ×100), expression of positive Alpha-fetoprotein(AFP) staining **(B)** (SP ×100) and positive glypican-3(GPC-3) staining **(C)** (SP ×100) in the liver metastases.

After one cycle of chemotherapy, tumor marker AFP rose from 300 to 1,931.90 ng/ml (**Table 1**). In order to clarify the nature of the liver lesions, liver biopsy was performed. Microscopic observation showed that the tumor cells were distributed in plate shape, and abundant blood sinus structures were seen, adenocarcinoma area and hepatoid differentiation area coexist, polygonal tumor cells with larger volume and eosinophilic cytoplasm can be seen in the hepatoid differentiation area (**Figure 4A**). Liver biopsy pathology showed adenocarcinoma, poorly differentiated; IHC showed cancer cell expression Hept1 (-), CK19 (+++), CK7 (-), CDX-2 (focal +), Ki67 (70%+), CK20 (-), Villin (+++), Syn (focal +), CgA (focal +), CD56 (focal +); AFP (+), GPC-3 (+) (**Figures 4B, C**).

Combining the abnormal increase of serum AFP, microscopic pathological morphology and IHC features of tissue samples from presurgical EUS-FNS, surgery, and liver biopsy, this is a rare case of HAC of the duodenum with liver metastasis.

However, the disease progressed rapidly, and the patient died within 1 month after biopsy. The survival time was only 161 days. Patients and their families hope that the information they provide can help clinicians have a deeper understanding of this kind of diseases, thus helping more patients.

## Discussion

This is a case report about the rare, highly malignant duodenum HAC. Because of the highly invasive and rapid progress nature of HAC, early diagnosis is particularly important. The diagnosis of HAC mainly depends on the pathological morphology; whatever the origin, most of them show similar morphological characteristics. Both adenocarcinoma and hepatoid differentiation areas can be observed in HAC. Adenocarcinoma areas are tubular in shape, and hepatoid differentiation areas have a similar arrangement to that of primary liver cancer. Furthermore, tumor cells in hepatoid differentiation areas have rich, eosinophilic or transparent staining cytoplasm, the nucleus is centered, mostly (quasi) circular or vacuolar, the nucleolus is large and obvious, mitotic images can be seen, and some of them have flake necrosis (21). Some scholars believe that the diagnosis of HAC can be established as long as the hepatoid differentiation area is found pathologically (22). However, the hepatoid differentiation area of HAC is mostly located in the deep part of the tumor, which makes the diagnosis extremely difficult. Important IHC markers included AFP, GPC-3, SALL4, and HSP70, among which the positive rate of AFP and GPC-3 was 85%~95% and 100%, respectively (22, 23). It is generally believed that AFP and GPC-3 are more specific markers in identifying hepatocyte differentiation areas and have been used as markers for the diagnosis of HAC routinely (24). In an analysis of 42 HAC cases reported by Zeng et al. (8), serum AFP levels were elevated in most patients with HAC, usually detected at extremely high levels (>1,000 ng/ml), while other serological biomarkers such as CEA and CA19-9 were consistently within the normal range, which were consistent with the clinical features of this patient. Abnormal elevation of serum AFP contributes to the objective diagnosis of the disease; however, the absence of a significant elevation of AFP could also be seen in some cases. The imaging features of HAC were not specific either, making it difficult to distinguish from other liver metastases (9). As HAC of the duodenum is rare and AFP staining is not included in routine IHC in CRC, it is difficult to conduct studies on large samples of this special type of tumor.

Gastric HAC is relatively more common than the others, so the research on this special type of tumor mostly focuses on gastric HAC. Clinical treatment of gastric HAC is usually preceded by radical gastrectomy. Radical gastrectomy combined with resection of liver metastases is feasible when liver metastasis is clearly identified and patient conditions permit. If radical resection of gastric lesions is not possible, surgical treatment is still recommended, and the surgical method should be changed to palliative gastrectomy. For patients with hepatic metastasis, palliative gastrectomy can be combined with other treatment methods, such as hepatic artery intubation chemotherapy (25). At present, there are no clear research results on the specific programs of postoperative adjuvant therapy (26). Simmet et al. (27) reported two cases of gastric HAC with hepatic metastasis, which achieved complete remission (CR) after first-line chemotherapy with cisplatin and etoposide, suggesting that cisplatin-based chemotherapy regimen may be the best choice for first-line chemotherapy for patients with gastric HAC. Due to the rarity of cases and lack of a large-sample randomized controlled study, the current clinical treatment strategy for patients with HAC of the duodenum is similar to that for CRC. Surgical treatment is the first choice, followed by chemotherapy and radiotherapy. It has been reported that the serum AFP level of HAC patients is correlated with prognosis. In most cases, the higher the level of serum AFP after the operation, the higher the rate of liver metastasis, and the worse prognosis (28). The reason may be that the increase of serum AFP can upregulate the expression of vascular endothelial growth factor receptor (VEGFR), which further leads to the formation of tumor emboli (29). However, whatever the level of AFP, the prognosis of patients with HAC is much worse than that of patients with normal adenocarcinoma. The average survival time is 10–18 months, and the 1-, 3-, and 5-year survival rates are 37.5%, 12.5%, and 8.3%, respectively (7).

Most of the previously reported cases of HAC had a primary lesion in the stomach, and most of the cases showed abnormal elevation of serum AFP in the preoperative period; whereas in this case, the primary lesion was located in the duodenum, which is a rare site, and the elevation of serum AFP was observed in the postoperative period, which made the diagnosis of this case less precise. The reason for reporting this case is to draw the clinician’s attention to the specific pathological type of HAC. When the pathological morphology of the tumor tissue tends to coexist with areas of adenocarcinoma and hepatoid differentiation, the possibility of HAC should be highly suspected and the accurate diagnosis should be identified as soon as possible. Moreover, the normal serum AFP at the time of diagnosis of the primary lesion cannot exclude the possibility of hepatocellular adenocarcinoma.

In conclusion, HAC of the duodenum is a rare special type of duodenal cancer with poor prognosis. It is often difficult to make a definite diagnosis in the early stage. The disease was not considered in the first EUS-FNA biopsy and pancreaticoduodenectomy; the diagnosis was made after the fast elevation of AFP and liver biopsy. It is suggested that for middle-aged and elderly patients, if the level of serum AFP increases and there is insufficient evidence for the diagnosis of hepatocellular carcinoma, HAC should be considered. Due to the low incidence rate, the treatment experience for this kind of disease is insufficient; targeted and effective standardized treatment remains to be explored.

In reviewing the paper, the following shortcomings remain, as there is no standard treatment regimen for this rare case, which is a common dilemma in the treatment of HAC. Focusing on this case, although the patient’s symptoms were somewhat relieved by the systemic treatment described above, it is undeniable that the patient’s survival was still relatively short and the exact mechanism of this is still to be explored in a pooled analysis of a large number of cases in order to find more effective treatment regimens.

## Data availability statement

The original contributions presented in the study are included in the article/supplementary material. Further inquiries can be directed to the corresponding author.

## Ethics statement

Written informed consent was obtained from the individual(s) for the publication of any potentially identifiable images or data included in this article.

## Author contributions

ND and LL provided ideas for this case. XW, ND, LL, and BL drafted the manuscript and provided figures. LL, XW, JH, and XQ acquired, analyzed, and interpreted the data. ND, LL, XQ, and BL revised the manuscript critically for important intellectual content and agreed to be accountable for all aspects of the work in ensuring that questions related to the accuracy or integrity of any part of the work are appropriately investigated and resolved. All authors contributed to the article and approved the submitted version manuscript.

## Funding

This study was supported by the Basic Research Project of Jiangsu Province (No. BK20211007).

## Conflict of interest

The authors declare that the research was conducted in the absence of any commercial or financial relationships that could be construed as a potential conflict of interest.

## Publisher’s note

All claims expressed in this article are solely those of the authors and do not necessarily represent those of their affiliated organizations, or those of the publisher, the editors and the reviewers. Any product that may be evaluated in this article, or claim that may be made by its manufacturer, is not guaranteed or endorsed by the publisher.
